# Effects of corn gluten feed inclusion at graded levels in a corn-soybean diet on the ileal and fecal digestibility of growing pigs

**DOI:** 10.1186/2049-1891-5-40

**Published:** 2014-08-20

**Authors:** Gerardo Mariscal Landín, Tércia Cesária Reis de Souza, Ericka Ramírez Rodríguez

**Affiliations:** 1 Centro Nacional de Investigación en Fisiología Animal, Instituto Nacional de Investigaciones Forestales Agrícolas y Pecuarias, Km 1, Carretera a Colón, Ajuchitlán Colón, Querétaro 76280, México; 2 Facultad de Ciencias Naturales, Universidad Autónoma de Querétaro, Santiago de Querétaro, Av. De las Ciencias s/n Juriquilla 76000, Querétaro, Querétaro, México

**Keywords:** Amino acids, Corn gluten feed, Energy, Ileal digestibility, Pigs, Total tract digestibility

## Abstract

**Background:**

This study aimed to determine the effect of the inclusion of corn gluten feed (CGF) on the apparent and standardized ileal digestibility of protein and amino acids and the apparent ileal and total tract digestibility of energy in growing pigs. The study was performed using 16 barrows (weight, 45.3 ± 4.5 kg) that were fitted with a T cannula at the terminal ileum. There were four treatments: a corn-soybean diet without CGF and three corn-soybean diets containing increasing levels of CGF (65, 130, and 195 g/kg). Data were analyzed according to a randomized complete block design, four blocks with four pigs each (one pig per treatment). The trend of the response (linear or quadratic) was determined using orthogonal contrasts, and when a linear effect was determined, a linear equation was obtained.

**Results:**

The results showed that the inclusion up to 195 g/kg of CGF in the corn-soybean diet did not diminish the ileal digestibility (apparent and standardized) of protein and amino acids (*P* > 0.05), except that of phenylalanine, cystine, and proline. A linear decrease (*P* < 0.05) per gram of CGF added to the diet in the apparent and standardized ileal digestibility of phenylalanine (0.011 and 0.015 percentage units, respectively), cystine (0.048 and 0.043 percentage units, respectively), and proline (0.045 and 0.047 percentage units, respectively) was noted. Similarly, ileal digestibility of dry matter and energy were adversely affected (reduced by 0.028 and 0.025 percentage units, respectively, per gram of CGF increment in the diet). A significant (*P* < 0.05) linear reduction in total tract digestibility with increase in CGF amount in the diet was observed for energy (0.027 percentage units), dry matter (0.027 percentage units), crude protein (0.020 percentage units), and neutral detergent fiber (0.041 percentage units) per gram of CGF added to the diet.

**Conclusion:**

CGF did not affect the ileal digestibility of protein and most amino acids but reduced the ileal and total tract digestibility of energy.

## Background

Corn (*Zea mays*) is the most harvested cereal worldwide [1]. Most of the corn produced is processed to extract flour, syrup, sweeteners, starches, oils, and ethanol. When wet milling is used to process corn, the germ is separated from the kernel, processed to obtain edible oil, and the resulting flour is mixed with the bran to produce corn gluten feed (CGF) [2], which has traditionally been considered a protein feed [3], although it is rich in fiber [4,5]. Therefore, the inclusion of CGF in pig diets has been limited, since fiber can affect directly [6,7] and indirectly [8,9] the digestibility of amino acids by increasing endogenous protein losses. Thus, this study aimed to determine the effects of inclusion of CGF in a corn-soybean meal diet on the apparent and standardized ileal digestibility of amino acids and apparent total tract digestibility of energy in growing pigs. We hypothesized that the inclusion of CGF decreases the ileal digestibility of amino acids and energy and the total tract digestibility of energy.

## Materials and methods

This study was approved by the Scientific Associate Technical Group Committee of CENID Physiology. The animals used in this study were cared for in accordance with the guidelines issued by the Mexican Official Standard for the Production, Protection and Use of Lab Animals [10] and the guidelines of the International Guiding Principles for Biomedical Research Involving Animals [11]. The study was performed at the experimental farm of CENID-Physiology.

### Animals

Sixteen barrows (Duroc × Landrace) weighing 45.3 ± 4.5 kg were used. The animals were placed in individual metabolic cages provided with a self-feeder and a low-pressure drinking nipple in a temperature controlled room at 20 ± 2°C. Animals were fed twice daily at 0800 h and 1800 h and had free access to water. A T cannula was fitted at the terminal ileum of each animal as previously described [12]. After surgery, therapeutic treatment (penicillin, 600,000 IU; streptomycin, 750 mg; oxytetracycline, 500 mg) was administered for 3 d; the post-surgery period lasted 21 d. From the second day after the post-surgery period, pigs began to receive 100 g of feed, which was increased by 100 g/day until the feed intake reached the level before surgery. During the experimental period the pigs were fed at 2.5-times the maintenance requirement of digestible energy of 460 kJ/kg BW^0.75^[13]. Pigs had free access to water.

### Experimental diets

The experimental diets were produced using corn, soybean meal, and CGF (Table 1). Four diets were formulated (Table 2) with increasing levels of CGF (0, 65, 130, and 195 g/kg of diet) at the expenses of corn and soybean meal. Corn oil was included at a rate of 40 g/kg. Salt, vitamins, and minerals were included at the levels that met or exceeded the National Research Council (NRC) nutritional requirements [5]; chromic oxide was added at a rate of 3 g/kg of feed as an inert marker [14]. The total fecal collection was performed daily for 5 d to achieve the required chromic oxide rate. Ferric oxide was added (3 g/kg of diet) to the first meal of the fifth day (to mark the start of the collection period) as well as to the first meal of the eleventh day (to mark the end of the collection period) [15].

**Table 1:** Chemical composition of raw materials as fed-basis (g/kg)

**Item**	**SBM**^**1**^	**YC**	**CGF**
Dry matter	899.5	900.3	891.2
Crude protein	468.1	108.5	208.4
NDF^2^	89.5	119.7	338.7
ADF^3^	57.4	45.0	106.5
Amino acids			
Alanine	24.8	7.2	17.1
Aspartic acid	67.5	9.7	17.3
Arginine	42.6	8.2	12.1
Cystine	8.3	2.7	4.3
Glutamic acid	111.7	20.8	45.3
Glycine	24.1	6.1	12.2
Histidine	10.1	4.7	7.1
Isoleucine	24.5	3.3	7.6
Leucine	44.2	10.1	23.8
Lysine	30.3	2.8	5.4
Methionine	7.8	2.0	3.4
Phenylalanine	28.9	4.7	9.7
Proline	31.1	8.8	16.6
Serine	30.7	5.7	11.5
Threonine	27.9	5.6	12.1
Tyrosine	20.8	3.9	8.1
Valine	25.5	5.6	12.1

^1^Raw materials: SBM = soybean meal, YC = yellow corn, CGF = corn gluten feed.

^2^NDF = neutral detergent fiber.

^3^ADF = acid detergent fiber.

**Table 2:** Composition and analyzed nutrient composition of experimental diets, as fed-basis (g/kg)

**g CGF/kg feed**	**0**	**65**	**130**	**195**
Yellow corn	762.9	716.9	671.2	625.6
Soybean meal	155.9	136.7	117.4	98
Corn gluten feed		65.7	131	196.4
Corn oil	40.0	40.0	40.0	40.0
Calcium carbonate	17.9	19.2	20.5	21.9
Dicalcium phosphate	10.4	8.4	6.3	4.1
Salt	3.5	3.5	3.5	3.5
L-Lysine HCl	2.5	2.4	2.4	2.3
Tryptosine	1.4	1.9	2.4	2.9
Threonine	0.2			
Vitamins^1^	1.6	1.6	1.6	1.6
Minerals^2^	0.7	0.7	0.7	0.7
Chromium oxide	3.0	3.0	3.0	3.0
Chemical analysis				
Dry matter	904.3	908.3	904.9	907.1
Protein	128.7	127.7	140.2	135.7
Energy, MJ	17.6	17.6	17.6	17.2
NDF	103.5	121.8	138.3	149.2
Amino acids				
Alanine	7.4	8.5	9.8	9.2
Arginine	8.8	9.8	11.0	9.6
Aspartic acid	13.5	14.0	15.5	13.3
Cystine	2.9	2.9	2.7	2.4
Glutamic acid	24.1	27.1	31.6	28.0
Glycine	6.6	7.2	8.1	7.4
Histidine	3.9	4.6	4.9	5.0
Isoleucine	2.6	4.3	5.3	5.0
Leucine	9.8	12.3	14.4	13.3
Lysine	9.3	9.8	9.8	9.4
Methionine	1.8	1.7	1.7	1.4
Phenylalanine	5.3	6.2	7.1	6.1
Proline	8.3	11.8	12.2	8.4
Serine	7.3	7.6	8.8	7.5
Threonine	6.4	6.9	7.8	7.1
Tyrosine	4.3	5.2	5.8	5.0
Valine	3.8	6.3	7.4	7.3

^1^Provided per kg piglet diet: Cl, 1.65 g; Na, 0.87 g; Cu, 7.7 mg; Fe, 89.25 mg; Mn, 19.98 mg; Se, 0.087 mg; I, 0.053 mg.

^2^Provided per kg piglet diet: vitamin A, 6600 IU; vitamin D, 660 IU; vitamin E, 100 IU; choline, 350 mg; niacin, 54 mg; pantothenic acid, 13.15 mg; riboflavin, 2.2 mg; B_12_, 36 μg.

NDF = neutral detergent fiber.

### Sample collection

The experimental period lasted 12 d; this included 5 d for adaptation to the diet, 5 d for the collection of feces, and 2 d for the collection of ileal digesta. Ileal digesta was collected in plastic bags (length, 11 cm; width, 5 cm) containing 10 mL of 0.2 mol/L solution of HCl to block any bacterial activity. Bags were attached to the barrel of the cannula by using a rubber band. Ileal digesta was collected continuously over the course of 12 h each day. When the bags were filled, they were transferred to a container and frozen at -20°C until lyophilization. All fecal samples were collected, frozen, and kept at -20°C. At the end of the experimental period, the feces were defrosted and homogenized to obtain 10% of the weight as a final sample for lyophilizing.

### Chemical analysis

Ileal digesta and feces samples were lyophilized and ground in a laboratory mill by using a 0.5-mm mesh (Arthur H. Thomas Co., Philadelphia, PA). Raw materials, experimental diets, ileal digesta, and feces were analyzed for dry matter (DM) and crude protein (CP) according to methods 934.01 and 976.05 of the Association of Official Agricultural Chemists (AOAC) [16]; neutral detergent fiber (NDF), according to van Soest [17]; and energy, by using an oxygen bomb calorimeter (model 1281; Parr, Moline, IL). Chromic oxide levels in the diets, ileal digesta, and feces were determined according to Fenton and Fenton [14]. Amino acid analysis was performed following method 994.12 of the AOAC [16]; samples were hydrolyzed in 6 mol/L HCl at 110°C for 24 h. Methionine and cystine were oxidized with performic acid before the analysis. The amino acid analysis was performed according to the method reported by Henderson et al. [18] by using a high-performance liquid chromatography (HPLC) model (1100; Hewlett Packard).

### Data analysis

Apparent ileal or total tract digestibility (AID or ATTD) were estimated using the equation proposed by Fan and Sauer [19].

$$\mathrm{AID}\;=\;\left[1-\left[\left(\mathrm{ID}\;\times \mathrm{AF}\right)/\left(\mathrm{AD}\;\times \mathrm{IF}\right)\right]\right]\times 100\text{,}$$

where AID% is the apparent (ileal or total) digestibility of a nutrient in the diet, *ID* is the marker concentration in the diet (mg/kg of DM), *AF* is the concentration of nutrient in the ileal digesta or feces (mg/kg of DM), *AD* is the concentration of the nutrient in the diet (mg/kg of DM), and *IF* is the marker concentration in the ileal digesta or feces (mg/kg of DM).

The standardized ileal digestibility (SID) was obtained using the formula proposed by Furuya and Kaji [20].

$$\mathrm{SID}\;=\mathrm{AID}\;+\;\left(\mathrm{Endogenous}\;/\mathrm{Dietary}\;\mathrm{Content}\right)$$

where *SID* is the standardized ileal digestibility of a nutrient, *AID* is the coefficient of apparent ileal digestibility of a nutrient, and *Endogenous* is the endogenous ileal losses of a nutrient in mg/kg of dry matter intake. The calculations were performed using endogenous values reported by Mariscal-Landin and Reis de Souza [21]. *Dietary Content* is the amount of nutrient consumed in mg/kg of dry matter intake.

### Statistical analysis

Data were analyzed according to a randomized complete block design [22] by using the general linear model (GLM) procedure of statistical analysis system (SAS) [23]: four blocks with four pigs each (one pig per treatment). Each pig was the experimental unit, and an alpha value of 0.05 was used to assess the significance. The trend of the response (linear or quadratic) was determined using orthogonal contrasts [22]. When a linear effect was determined, a linear equation was obtained using the regression (REG) procedure of SAS [23].

## Results

### Apparent ileal digestibility

The results of apparent ileal digestibility are shown in Table 3. The inclusion of CGF significantly reduced (*P* < 0.05) the AID of dry matter; there was a reduction of 5.7 percentage units between the diet without CGF and the diet containing 195 g of CGF (87.7% *vs.* 82.0%). This adverse effect was also observed in energy digestibility, which was reduced by 4.9 percentage units (from 88.8 to 83.9 in the diets with 0 or 195 g of CGF, respectively). The digestibility of phenylalanine, cystine and proline decreased linearly (*P <* 0.05) in response to CGF increment in the diet. The average reduction in ileal digestibility of amino acids was 0.031 percentage units per gram of CGF included in the diet. Cystine digestion was the most affected (0.048 percentage units) and phenylalanine, the least (0.011 percentage units). The digestibility of the other amino acids was not affected by the inclusion of CGF at the levels used in this study.

**Table 3:** Apparent ileal digestibility (AID) coefficients (%) of experimental diets

**g CGF/kg feed**	**0**	**65**	**130**	**195**	**SEM**
Dry matter^A^	87.7	83.8	82.8	82.0	0.66
Protein	84.9	81.4	81.7	80.7	0.98
Energy^A^	88.8	86.0	84.6	83.9	0.66
Amino acids					
Alanine	82.4	81.1	80.3	81.5	1.31
Arginine	93.1	91.9	91.0	89.5	0.86
Aspartic acid	86.1	83.1	83.1	81.5	1.05
Cystine^A^	86.1	85.1	81.8	76.7	1.22
Glutamic acid	88.7	88.1	87.7	86.9	0.92
Glycine	80.2	79.3	77.5	76.3	1.38
Histidine	87.5	86.7	85.7	85.7	0.91
Isoleucine	79.0	83.5	83.1	83.1	1.63
Leucine	87.4	87.4	87.4	86.9	1.03
Lysine	92.7	90.8	90.6	90.6	0.67
Methionine	83.3	82.4	82.1	78.2	1.20
Phenylalanine^A^	93.7	92.1	92.0	91.4	0.30
Proline^A^	87.5	89.1	83.9	79.6	1.27
Serine	85.5	82.8	82.7	81.1	1.18
Threonine	81.9	77.8	77.5	77.2	1.52
Tyrosine	88.2	88.1	87.3	85.9	0.94
Valine	79.8	85.2	84.1	83.5	1.57

^A^Linear effect (*P* < 0.05).

### Standardized ileal digestibility

The standardized ileal digestibility of amino acids is shown in Table 4. In general, the inclusion of CGF in the diet did not affect (*P* > 0.05) the SID. However, a linear decrease in SID of phenylalanine (0.015 percentage units; from 98.7 to 95.8), cystine (0.043 percentage units, from 91.4 to 83.1), and proline (0.047 percentage units, from 103.7 to 94.9) was noted per gram inclusion of CGF. There was no significant reduction in the digestibility of the other amino acids (*P* > 0.05).

**Table 4:** Standardized ileal digestibility (SID) coefficients (%) of experimental diets

**g CGF/kg feed**	**0**	**65**	**130**	**195**	**SEM**
Protein	93.7	90.3	89.9	89.1	0.98
Amino acids					
Alanine	87.6	85.7	84.3	85.7	1.31
Aspartic acid	91.2	88.0	87.4	86.6	1.05
Arginine	97.1	95.5	94.7	92.7	0.86
Cystine^A^	91.4	90.3	87.3	83.1	1.22
Glutamic acid	91.9	91.0	90.1	89.7	0.92
Glycine	92.0	89.9	86.9	86.8	1.38
Histidine	91.8	90.3	89.2	89.1	0.91
Isoleucine	90.3	90.5	89.0	88.9	1.63
Leucine	92.5	92.0	90.9	90.6	1.03
Lysine	96.8	94.2	94.2	93.5	0.67
Methionine	87.2	86.2	86.6	83.2	1.20
Phenylalanine^A^	98.7	96.5	95.8	95.8	0.30
Proline^A^	103.7	100.5	95.5	94.9	1.27
Serine	92.4	89.3	88.4	87.9	1.18
Threonine	90.8	86.0	85.6	84.5	1.52
Tyrosine	91.4	90.7	89.7	88.7	0.94
Valine	90.3	91.6	89.5	89.0	1.57

^A^Linear effect (*P* < 0.05).

### Apparent total tract digestibility

The ATTD of dry matter, protein, energy, and NDF diminished (*P <* 0.05) linearly with an increase in CGF concentration in the diet (Table 5). The effect of CGF inclusion on ATTD was net and clear. The reduction of digestibility of dry matter and energy was 6.7 percentage units; for protein, 6.3 percentage units; and for NDF, 11.3 percentage units. The linear equations are shown in Table 6; a decrease of 0.02 to 0.03 percentage units in the ATTD of DM, CP, and energy per gram of CGF included in the diet and a decrease of 0.041 percentage units in the ATTD of NDF were noted per gram of CGF included in the diet.

**Table 5:** Apparent total tract digestibility (ATTD) coefficients (%) of experimental diets

**g CGF/kg feed**	**0**	**65**	**130**	**195**	**SEM**
Dry matter^A^	88.6	81.3	83.7	81.9	0.36
Protein^A^	86.9	76.9	83.4	80.6	0.59
Energy^A^	88.7	81.4	84.1	82.0	0.36
NDF^A^	62.5	47.8	55.2	51.2	1.42

^A^Linear effect (*P* < 0.05).

NDF = neutral detergent fiber.

**Table 6:** Linear relationships between corn gluten feed (CGF) inclusion and ileal and total tract digestibility coefficients

**Apparent ileal digestibility**		
Dry matter	DM = 86.77 (±1.02) – 0.028 (±0.008) gCGF^1^	*r*^2^ = 0.86
Energy	E = 88.22 (±0.96) – 0.025 (±0.008) gCGF	*r*^2^ = 0.92
Phenylalanine	Phe = 93.31 (±0.46) – 0.011 (±0.004) gCGF	*r*^2^ = 0.84
Cystine	Cys = 87.15 (±1.90) – 0.048 (±0.016) gCGF	*r*^2^ = 0.92
Proline	Pro = 89.36 (±1.89) – 0.045 (±0.016) gCGF	*r*^2^ = 0.78
**Standardized ileal digestibility**		
Phenylalanine	Phe = 98.13 (±0.51) – 0.015 (±0.004) gCGF	*r*^2^ = 0.78
Cystine	Cys = 92.19 (±1.88) – 0.043 (±0.015) gCGF	*r*^2^ = 0.94
Proline	Pro = 103.20 (±1.84) – 0.047 (±0.015) gCGF	*r*^2^ = 0.93
**Apparent total tract digestibility**		
Dry matter	DM = 86.52 (±1.16) – 0.027 (±0.010) gCGF	*r*^2^ = 0.47
Protein	CP = 83.85 (±1.74) – 0.020 (±0.014) gCGF	*r*^2^ = 0.14
Energy	E = 86.64 (±1.20) – 0.027 (±0.010) gCGF	*r*^2^ = 0.46
NDF	NDF = 58.13 (±2.97) – 0.041 (±0.015) gCGF	*r*^2^ = 0.29

^1^gCGF = grams of corn gluten feed added to the diet.

NDF = neutral detergent fiber.

## Discussion

Fiber has been defined as “vegetal compounds, from carbohydrate nature and resistant to digestive enzymes”; it is classified according to solubility as soluble fiber (*β*-glucans, gums, and mucilages) and insoluble fiber (cellulose and most hemicelluloses) [24]. CGF is a mixture of corn structures that remain after the removal of most starch and gluten germ [2]. Approximately, two-thirds of these corn structures are fibrous structures, and one-third consists of soluble compounds [25]. The fiber obtained from corn is essentially insoluble (99% of the total fiber) and represents over 30% of the dry matter [25]; the CGF used in this study had 338.7 g NDF/kg. Insoluble fiber is known to cause endogenous protein losses and reduce the AID of protein and amino acids because it increases mucin secretion as well as mucosal cell shedding via its abrasive effect [8,26,27]. The protein content of CGF is not contained in the fibrous part (bran). It is present in the soluble fraction that is mixed with bran at the end of the milling process [2]; therefore, fiber does not block the enzyme access to proteins as is noted in wheat bran, in which a high proportion of protein is present in the aleurone cells [28]. Furthermore, several authors [29-31] have suggested that insoluble fiber has a minor effect on the ileal digestibility of amino acids and protein. Both findings (protein is not present in the fibrous part and the minor effect of insoluble fiber on the ileal digestibility of protein) could explain the almost null effect of CGF on the ileal digestibility of protein and amino acids. The decrease in the ileal digestibility of proline could be because of the proteins present in the fibrous fraction; these proteins are rich in extensine—a proline-rich protein that resembles collagen—and are closely linked to the cellulose fraction [32-34]. The negative effect on the ileal digestibility of dry matter and energy was due to an increase in the levels of non-digestible compounds (insoluble fiber) after treatment with CGF; as mentioned before, two-thirds of the CGF is insoluble fiber, and hence, it dilutes the digestible dry matter and energy as has been reported previously [35].

In the large intestine, the undigested food (mainly non-starch polysaccharides and protein) is fermented by microorganisms [36]. This is a long process, but the insoluble fiber decreases the residence time of digesta in the cecum and colon, and this effect is related to the fiber content of the diet [24,29,35]. Insoluble fibers are resistant to fermentation; therefore, they play a major role in fecal bulking, unlike soluble fibers that are almost completely fermented and have little effect on increasing fecal bulk [37]. This limited fermentation of insoluble fibers by intestinal bacteria reduces the total digestibility of protein and energy [31,35,38]; consequently, the digestible energy is low in diets containing CGF. Furthermore, fibers increase the production of mucin, an almost indigestible protein, thereby increasing protein excretion in the feces [39] and lowering protein digestion. As discussed before, the inclusion of CGF in diets has a mild effect on the ileal digestibility of amino acids; however, it adversely affects energy digestibility. Few studies have quantitatively investigated the adverse effect of NDF on energy digestibility; the findings reported in this study are in agreement with those reported by Dégen [31]. However, in sows, fiber plays an important role in avoiding stereotyped conducts, and thus, sows are able to obtain more energy from fiber than growing pigs [24,40,41].

## Conclusions

CGF did not affect the apparent and standardized ileal digestibility of protein and most amino acids, except that of phenylalanine, cystine, and proline. However, it linearly decreased the ileal digestibility of energy and the total tract digestibility of protein and energy.

## Abbreviations

CGF: Corn gluten feed; AID: Apparent ileal digestibility; SID: Standardized ileal digestibility; ATTD: Apparent total tract digestibility; NDF: Neutral detergent fiber.

## Competing interests

The authors declare that they have no competing interests.

## Authors’ contributions

GML conceived and designed the study; ERR carried out laboratory analysis; TCRS contributed to data analysis; GML and TCRS drafted the manuscript. All authors read and approved the final manuscript.
